# Dietary and circulating butyrate are independently associated with kidney function in diabetes: a dual-cohort analysis

**DOI:** 10.3389/fnut.2025.1671238

**Published:** 2025-09-29

**Authors:** Leying Zhao, Cong Zhao, Aoshuang Li, Qinyang Gao, Sinan Ai, Yaoxian Wang, Zhenjie Chen, Zhen Wang

**Affiliations:** ^1^Dongzhimen Hospital, Beijing University of Chinese Medicine, Beijing, China; ^2^Beijing University of Chinese Medicine, Beijing, China; ^3^South Area, Guang'anmen Hospital, China Academy of Chinese Medical Sciences, Beijing, China; ^4^Lintong Rehabilitation and Convalescent Center of PLA Joint Logistic Support Force, Xi'an, China; ^5^China-Japan Friendship Hospital, Beijing, China; ^6^Renal Research Institution of Beijing University of Chinese Medicine, Beijing, China; ^7^Henan University of Chinese Medicine, Zhengzhou, China

**Keywords:** butyrate, diabetic kidney disease, gut-kidney axis, renal function, short-chain fatty acids

## Abstract

**Background:**

The gut microbiota-derived metabolite butyrate has been implicated in maintaining renal homeostasis through anti-inflammatory and immunomodulatory pathways. However, evidence from large-scale human studies, especially in high-risk diabetic populations, remains limited. This study aimed to investigate the association between butyrate exposure and renal function in adults with diabetes, using a dual-cohort design.

**Methods:**

We analyzed data from 7,723 adults with diabetes across ten NHANES cycles (1999–2018) to evaluate the association of dietary butyrate intake with estimated glomerular filtration rate (eGFR) and albuminuria. Multivariable linear regression, restricted cubic spline modeling, and subgroup analyses were performed with survey weighting. For external validation, we recruited a Chinese cohort of 70 patients with diabetic kidney disease (DKD) and measured serum butyrate and isobutyrate concentrations using UPLC-MS/MS. Associations with eGFR and 24-h urinary protein were assessed using adjusted regression models.

**Results:**

In the NHANES cohort, higher dietary butyrate intake was independently associated with a higher eGFR (*β* = 1.61; 95% CI: 0.29–2.92; *p* = 0.02), with a significant nonlinear dose–response (*P* for non-linearity = 0.0006). No significant associations were found with albuminuria. In the Chinese cohort, serum butyrate was positively associated with eGFR (*β* = 0.05; 95% CI: 0.01–0.08; *p* = 0.02), but not with proteinuria. Serum isobutyrate also showed a positive association with eGFR (*β* = 0.15; 95% CI: 0.02–0.28; *p* = 0.02). Sensitivity analyses confirmed the robustness of these findings among participants with both diabetes and CKD.

**Conclusion:**

This dual-cohort study provides the first epidemiological evidence that higher levels of butyrate—whether from dietary intake or serum concentration—are independently associated with better renal function in adults with diabetes. These findings underscore the relevance of the gut-kidney axis in diabetic kidney disease and suggest that enhancing endogenous butyrate production through diet or microbiota-targeted strategies may offer a novel avenue for renoprotection.

## 1 Introduction

Chronic kidney disease (CKD) has emerged as a pressing global health concern (1), with its etiological profile shifting from traditional causes such as hypertension and glomerulonephritis to metabolic disorders—particularly diabetes (2). Diabetic kidney disease (DKD) is now the leading cause of end-stage renal disease (ESRD) (3), placing a substantial burden on healthcare systems and significantly compromising patient quality of life. Recent global estimates suggest that approximately 40% of the 589 million individuals living with type 2 diabetes will develop CKD (4), with the risk rising progressively alongside disease duration (5). These alarming trends underscore the urgent need for effective primary prevention strategies.

In recent years, the “gut-kidney axis” has gained prominence as a conceptual framework for understanding the pathophysiology of CKD (6). Gut dysbiosis and its microbial metabolites are increasingly implicated in renal injury. Disruption of the intestinal barrier permits the translocation of uremic toxins—such as indoxyl sulfate—into systemic circulation, initiating inflammatory and oxidative stress pathways (7) that exacerbate glomerular fibrosis and accelerate renal function decline. Central to this axis are short-chain fatty acids (SCFAs), particularly butyrate, which have been the focus of growing scientific interest due to their diverse biological activities. Butyrate, a key fermentation product of dietary fiber, serves not only as a primary energy source for colonocytes (8) but also exhibits anti-inflammatory and immunoregulatory effects through the activation of G-protein-coupled receptors (GPRs) (9) and the inhibition of histone deacetylases (HDACs) (10), supporting its potential role as a reno-protective metabolite. In contrast, isobutyrate, another SCFA analyzed in our study, is primarily derived from the microbial fermentation of branched-chain amino acids like valine (11). While both fatty acids are implicated in metabolic health, the biological roles of isobutyrate are less characterized than those of butyrate (12). However, emerging evidence suggests it may also possess anti-inflammatory properties and serve as a marker of altered protein metabolism and specific gut microbial activities (13, 14).

While preclinical evidence from animal and *in vitro* studies consistently demonstrates the reno-protective effects of butyrate (15, 16), supporting data from human populations remain limited (17, 18). Existing epidemiological studies have largely focused on total dietary fiber intake, providing only indirect insights into SCFA-mediated mechanisms (19). Moreover, studies that directly quantify circulating SCFA concentrations are often constrained by small sample sizes (18) and rarely focus on diabetic populations—despite their elevated risk for renal complications. Consequently, the independent association between butyrate levels—whether derived from dietary intake or measured in serum—and key indicators of renal function such as estimated glomerular filtration rate (eGFR) and albuminuria remains poorly defined, representing a critical knowledge gap.

To address this gap, the present study adopts a dual-cohort design to systematically investigate the relationship between butyrate and renal function. First, using nationally representative data from the U. S. National Health and Nutrition Examination Survey (NHANES), we examine the association between dietary butyrate intake and both eGFR and albuminuria in adults with diabetes. Second, in a clinically characterized cohort of patients with confirmed DKD, we analyze serum concentrations of butyrate and isobutyrate in relation to renal function metrics. The primary objective is to determine whether higher butyrate exposure is independently associated with better renal function, thereby offering crucial population-based evidence for its potential role in the pathogenesis and prevention of DKD.

## 2 Methods

### 2.1 Study design and population

Data for this study were drawn from ten consecutive cycles of NHANES conducted between 1999 and 2018. NHANES, administered by the U. S. Centers for Disease Control and Prevention (CDC), adopts a multistage, stratified probability sampling framework to generate a nationally representative dataset. It encompasses comprehensive information on demographics, dietary habits, physical and laboratory assessments, and health-related questionnaires, making it a critical resource for epidemiological research on chronic diseases.

**Figure 1:**
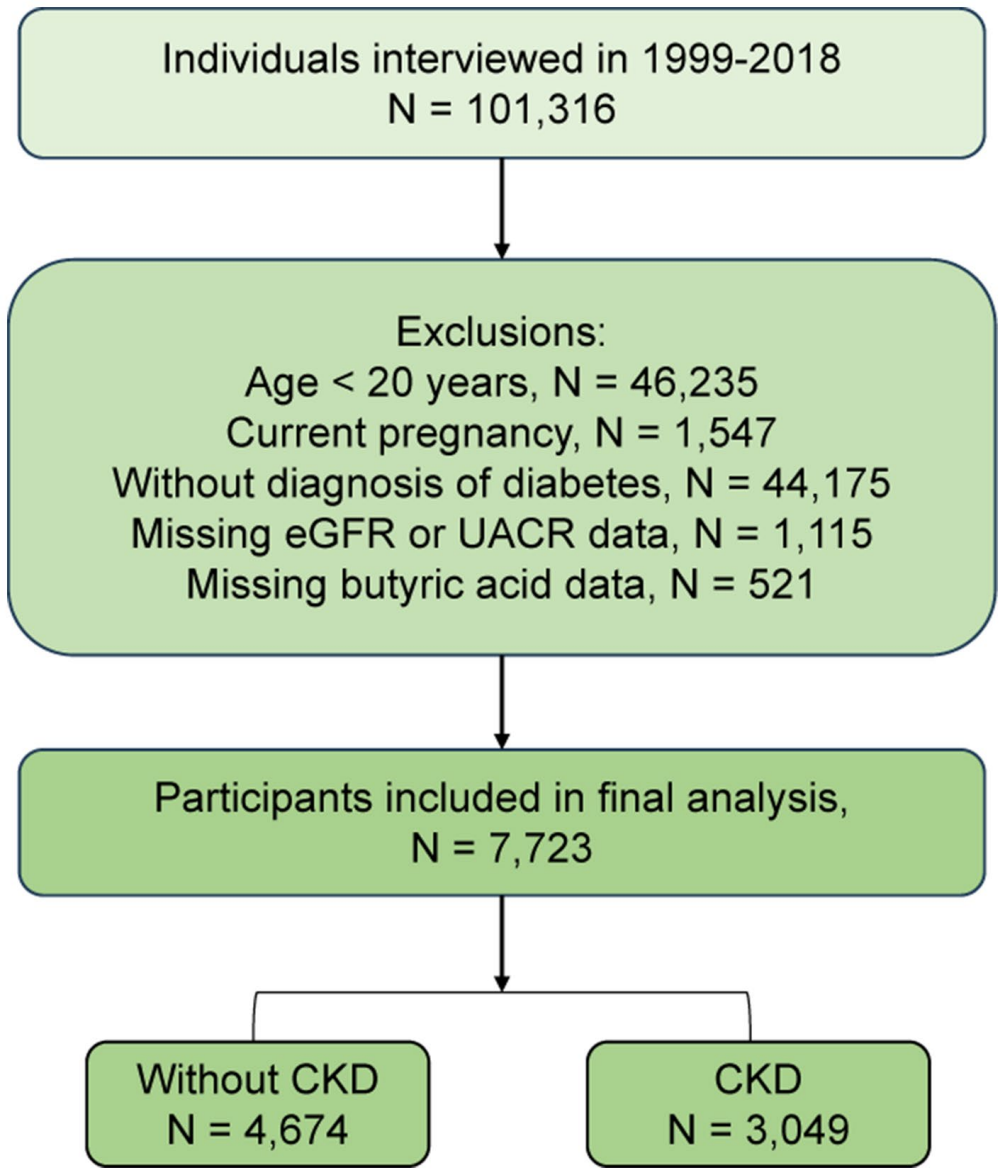
Flowchart of participant selection from the National Health and Nutrition Examination Survey. eGFR: Estimated glomerular filtration rate; UACR, urinary albumin-to-creatinine ratio.

The participant selection process is illustrated in Figure 1. From an initial pool of 101,316 individuals, we excluded those aged <20 years (*n* = 46,235), pregnant participants (*n* = 1,547), individuals not meeting diabetes diagnostic criteria (*n* = 44,175), those missing data on eGFR or urinary albumin-to-creatinine ratio (UACR) (*n* = 1,115), and participants without dietary butyrate intake data (*n* = 521). The final analytic sample comprised 7,723 eligible individuals. All participants provided written informed consent, and the NHANES protocol was approved by the National Center for Health Statistics (NCHS) Research Ethics Review Board; thus, no additional institutional approval was required.

Diabetes was defined by any of the following: self-reported diagnosis, HbA1c > 6.5%, fasting plasma glucose ≥7.0 mmol/L, random plasma glucose ≥11.1 mmol/L, a 2-h oral glucose tolerance test (OGTT) result ≥11.1 mmol/L, or use of antidiabetic medication (20). CKD was defined as an eGFR <60 mL/min/1.73 m^2^ or a UACR >30 mg/g, in accordance with clinical guidelines (21).

For external validation, a Chinese cohort of patients with DKD was recruited from Dongzhimen Hospital, Beijing University of Chinese Medicine, between May and December 2024. Ethical approval was granted by the institutional ethics committee (Approval No. 2024DZMEC-098-01), and written informed consent was obtained from all participants.

Inclusion criteria were: (1) age 30–90 years; (2) diagnosis of DKD in accordance with the 2007 NKF-KDOQI guidelines (22), the 2022 KDIGO Clinical Practice Guideline (23), and the 2021 Chinese guidelines for DKD management (24); (3) availability of complete clinical data; and (4) informed consent. Exclusion criteria were: (1) history of dialysis or kidney transplantation; (2) primary or secondary nephropathy unrelated to DKD; (3) current urinary tract infection or systemic inflammation; (4) severe hepatic dysfunction, autoimmune or psychiatric disorders, malignancy, or hematological disease; and (5) major trauma or surgery within the preceding 6 months.

### 2.2 Exposure assessment

In the NHANES cohort, dietary intake was evaluated using 24-h dietary recall interviews. Each participant completed at least one recall, conducted in person at a Mobile Examination Center (MEC), and a second via telephone within 3 to 10 days. Reported food items were mapped to the U. S. Department of Agriculture’s (USDA) Food and Nutrient Database for Dietary Studies (FNDDS) to estimate daily butyrate intake. The mean value of both recalls was used when available; otherwise, the single recall was utilized.

In the Chinese cohort, fasting venous blood samples were collected the morning after admission. Following centrifugation at 3,000 rpm for 10 min at 4 °C, the resulting serum was promptly aliquoted into Eppendorf tubes and stored at −80 °C until UPLC-MS/MS analysis. Serum concentrations of butyrate and isobutyrate were quantified using ultra-performance liquid chromatography–tandem mass spectrometry (UPLC-MS/MS) with electrospray ionization (ESI). Sample preparation involved protein precipitation with 200 μL of cold acetonitrile (containing [2H9]-valeric acid and [2H11]-hexanoic acid) mixed with 50 μL serum, followed by centrifugation (12,000 rpm, 10 min, 4 °C). An 80 μL aliquot of the supernatant was derivatized with 3-nitrophenylhydrazine (3-NPH) and N-(3-dimethylaminopropyl)-N′-ethylcarbodiimide (EDC) at 40 °C for 30 min. Chromatographic separation was performed using a Waters ACQUITY I-Class UPLC with a BEH C18 column (100 × 2.1 mm, 1.7 μm), using a mobile phase of 0.1% formic acid in water (A) and acetonitrile/methanol (2:1, v/v) (B). A linear gradient from 25 to 55% B over 9 min was applied at a flow rate of 0.35 mL/min. Mass detection was carried out using an AB SCIEX QTRAP 5500 in negative electrospray ionization (ESI) mode with multiple reaction monitoring (MRM). Quantification was performed using the internal standard method in SCIEX OS-MQ software. Representative chromatograms demonstrating the typical metabolic profile and the analytical platform’s stability are provided in Supplementary Figure S1.

### 2.3 Outcome variables

The primary outcome measures were eGFR and proteinuria. eGFR was calculated using the 2021 CKD-EPI creatinine equation based on serum creatinine, age, and sex. In the NHANES cohort, proteinuria was assessed using the UACR from a spot urine sample. In the Chinese cohort, total 24-h urinary protein was measured.

### 2.4 Covariates

A comprehensive set of covariates was selected to account for potential confounding in the regression models. In NHANES, demographic variables (age, sex, race/ethnicity, marital status), lifestyle factors (smoking status, alcohol use, BMI), and comorbidities (hypertension, hyperlipidemia) were included. Hypertension was defined as systolic BP ≥ 140 mmHg, diastolic BP ≥ 90 mmHg, physician diagnosis, or antihypertensive medication use. Hyperlipidemia was defined by any of the following: triglycerides ≥150 mg/dL, total cholesterol ≥200 mg/dL, LDL-C ≥ 130 mg/dL, HDL-C ≤ 40 mg/dL (men) or ≤50 mg/dL (women), or use of lipid-lowering therapy. In the Chinese cohort, covariates included age, sex, and fasting blood glucose measured at baseline.

### 2.5 Statistical analysis

Descriptive statistics were used to summarize baseline characteristics. For the NHANES cohort, all analyses accounted for the complex survey design by using appropriate sample weights. Data are presented as weighted mean ± standard error (SE) for continuous variables and as unweighted counts (*n*) with weighted percentages (%) for categorical variables. For the Chinese cohort, which did not involve complex weighting, data are presented as mean ± standard deviation (SD) or median with interquartile range (IQR) for continuous variables, as appropriate, and as frequencies (*n*) with percentages (%) for categorical variables.

Multivariable linear regression models were used to assess associations between butyrate levels (dietary in NHANES; serum in Chinese cohort) and renal outcomes (eGFR and UACR or 24-h protein). For NHANES, weighted ordinary least squares (OLS) regression was applied. Model 1 adjusted for demographics and lifestyle factors; Model 2 additionally controlled for hypertension and hyperlipidemia. In the Chinese cohort, Model 1 adjusted for age and sex; Model 2 further included fasting glucose. Results were presented as regression coefficients (*β*) with 95% confidence intervals (CIs) and *p*-values.

To assess the dose–response relationship, butyrate levels were analyzed in two ways. As a continuous variable, we evaluated the linear association between each unit increase in butyrate and renal function outcomes. As a categorical variable, we divided participants into quartiles (Q1-Q4) to compare the outcomes of groups with progressively higher exposure levels against a reference group. In this context, the lowest quartile (Q1) served as the reference, while the upper quartiles (e.g., Q3 and Q4) represented cohorts with ‘higher levels’ of butyrate. A *P* for trend was calculated across these quartiles to test for a dose–response trend. In NHANES, subgroup and interaction analyses were performed to examine effect modification by age, sex, BMI, and comorbidities.

A sensitivity analysis that restricted the NHANES sample to participants with confirmed diabetes and CKD was conducted to evaluate robustness and explore potential reverse causality. All analyses were conducted using R software (version 4.4.2, R Foundation for Statistical Computing, Vienna, Austria), with statistical significance defined as a two-sided *p*-value < 0.05.

## 3 Result

### 3.1 Baseline characteristics of study participants

The final analysis included two independent cohorts. The NHANES cohort consisted of 7,723 individuals with diabetes (Table 1), with a mean age of 59.00 ± 0.25 years. Females accounted for 48.26% of participants, and 63.51% identified as non-Hispanic White. The mean eGFR was 85.30 ± 0.34 mL/min/1.73 m^2^, and the mean UACR was 116.66 ± 7.32 mg/g, indicative of significant albuminuria. The average daily dietary butyrate intake was 0.49 ± 0.01 g. Among this cohort, 3,049 individuals (39.48%) met the diagnostic criteria for CKD. Compared to participants without CKD, those with CKD were older, had lower educational attainment and household income, and were more likely to be unmarried or living alone. Furthermore, this group had a higher prevalence of smoking, elevated HbA1c levels, a greater burden of hypertension, and notably lower butyrate intake.

**Table 1:** Baseline characteristics of participants in NHANES.

	Total (*n* = 7,723)	Without CKD (*n* = 4,674)	With CKD (*n* = 3,049)	*p* value
Age, years	59.00 ± 0.25	56.48 ± 0.28	63.74 ± 0.37	< 0.0001
Sex, %				0.69
Female	3,706 (48.26)	2,271 (48.01)	1,435 (48.72)	
Male	4,017 (51.74)	2,403 (51.99)	1,614 (51.28)	
Ethnicity/Race, %				< 0.001
White	2,896 (63.51)	1724(64. 59)	1,172 (61.48)	
Black	1853 (13.74)	1,038(12.53)	815 (16.03)	
Mexican American	1,602 (9.20)	999 (9.30)	603 (9.02)	
Other races	1,372 (13.54)	913 (13.58)	459 (13.46)	
Marital status, %				< 0.0001
Married or living with a partner	4,635 (63.64)	2,971 (67.62)	1,664 (57.61)	
Not married nor living with a partner	3,034 (35.60)	1,665 (32.38)	1,369 (42.39)	
Education level, %				< 0.0001
Below high school	2,852 (24.91)	1,584 (21.75)	1,268 (30.91)	
High school and above	4,862 (75.01)	3,086 (78.25)	1776 (69.09)	
Alcohol user, %				< 0.0001
Yes	3,831 (55.74)	2,496 (61.14)	1,335 (51.14)	
Former	2,290 (26.32)	1,244 (24.81)	1,046 (31.75)	
No	1,289 (14.61)	742 (14.04)	547 (17.11)	
Smoker, %				< 0.001
Now	1,262 (16.50)	789 (16.79)	473 (15.97)	
Former	2,641 (34.65)	1,481 (32.50)	1,160 (38.77)	
Never	3,813 (48.78)	2,402 (50.71)	1,411 (45.26)	
BMI, kg/m^2^	32.86 ± 0.14	32.92 ± 0.17	32.75 ± 0.23	0.55
Butyric acid intake, g/day	0.49 ± 0.01	0.51 ± 0.01	0.46 ± 0.01	0.004
HbA1c, %	7.15 ± 0.03	6.98 ± 0.03	7.45 ± 0.04	< 0.0001
eGFR, mL/min/1.73 m^2^	85.30 ± 0.34	92.49 ± 0.33	71.81 ± 0.73	< 0.0001
Urinary albumin, mg/L	118.65 ± 7.66	11.81 ± 0.21	319.27 ± 21.52	< 0.0001
UACR, mg/g	116.66 ± 7.32	10.13 ± 0.11	316.70 ± 21.04	< 0.0001
Hypertension, %	5,532 (69.46)	3,037 (63.54)	2,495 (80.58)	< 0.0001
Hyperlipidemia, %	6,761 (88.66)	4,043 (88.06)	2,718 (89.78)	0.05

In contrast, the Chinese cohort comprised 70 patients with type 2 diabetic kidney disease (Table 2). This group was older (mean age: 60.91 ± 11.77 years) and had a lower proportion of females (37.14%). Their clinical characteristics reflected more advanced renal dysfunction, with a median eGFR of 30.17 (IQR: 11.97–72.52) mL/min/1.73 m^2^, serum creatinine of 229.75 (IQR: 107.50–450.03) μmol/L, and blood urea nitrogen of 14.11 (IQR: 8.98–24.34) mmol/L. The median UACR was markedly elevated at 1571.20 (IQR: 408.70–2850.30) mg/g. The mean fasting glucose was 8.73 ± 4.60 mmol/L, and the average uric acid concentration was 405.58 ± 91.47 μmol/L. Mean serum levels of butyrate and isobutyrate were 526.63 ± 193.85 ng/mL and 136.78 ± 55.69 ng/mL, respectively.

**Table 2:** Baseline characteristics of the study participants in Chinese corhort.

	Total (*n* = 70)	Female (*n* = 26)	Male (*n* = 44)	*p* value
Age, years	60.91 ± 11.77	64.77 ± 11.10	58.64 ± 11.68	0.03
Fasting plasma glucose, mmol/L	8.73 ± 4.60	9.06 ± 4.51	8.54 ± 4.69	0.65
eGFR, mL/min/1.73 m^2^	30.17 (11.97, 72.52)	48.18 (16.41, 74.47)	18.62 (10.41, 67.38)	0.25
Blood urea nitrogen, mg/dL	14.11 (8.98, 24.34)	11.79 (8.36, 22.09)	15.82 (9.53, 26.16)	0.25
Serum creatinine, μmoI/L	229.75 (107.50, 450.03)	138.20 (96.05, 360.78)	326.20 (111.48, 511.98)	0.02
UACR, mg/g	1571.20 (408.70, 2850.30)	1156.15 (192.20, 2694.70)	1800.25 (725.85, 2843.93)	0.70
UA, μmol/L	405.58 ± 91.47	375.99 ± 85.62	422.78 ± 91.28	0.04
Serum butyric acid (ng/mL)	526.63 ± 193.85	566.14 ± 223.14	503.29 ± 172.77	0.22
Serum isobutyric acid (ng/mL)	136.78 ± 55.69	145.46 ± 60.55	131.65 ± 52.65	0.34

### 3.2 Association between dietary butyrate and renal function in the NHANES cohort

#### 3.2.1 Association with eGFR

**Figure 2:**
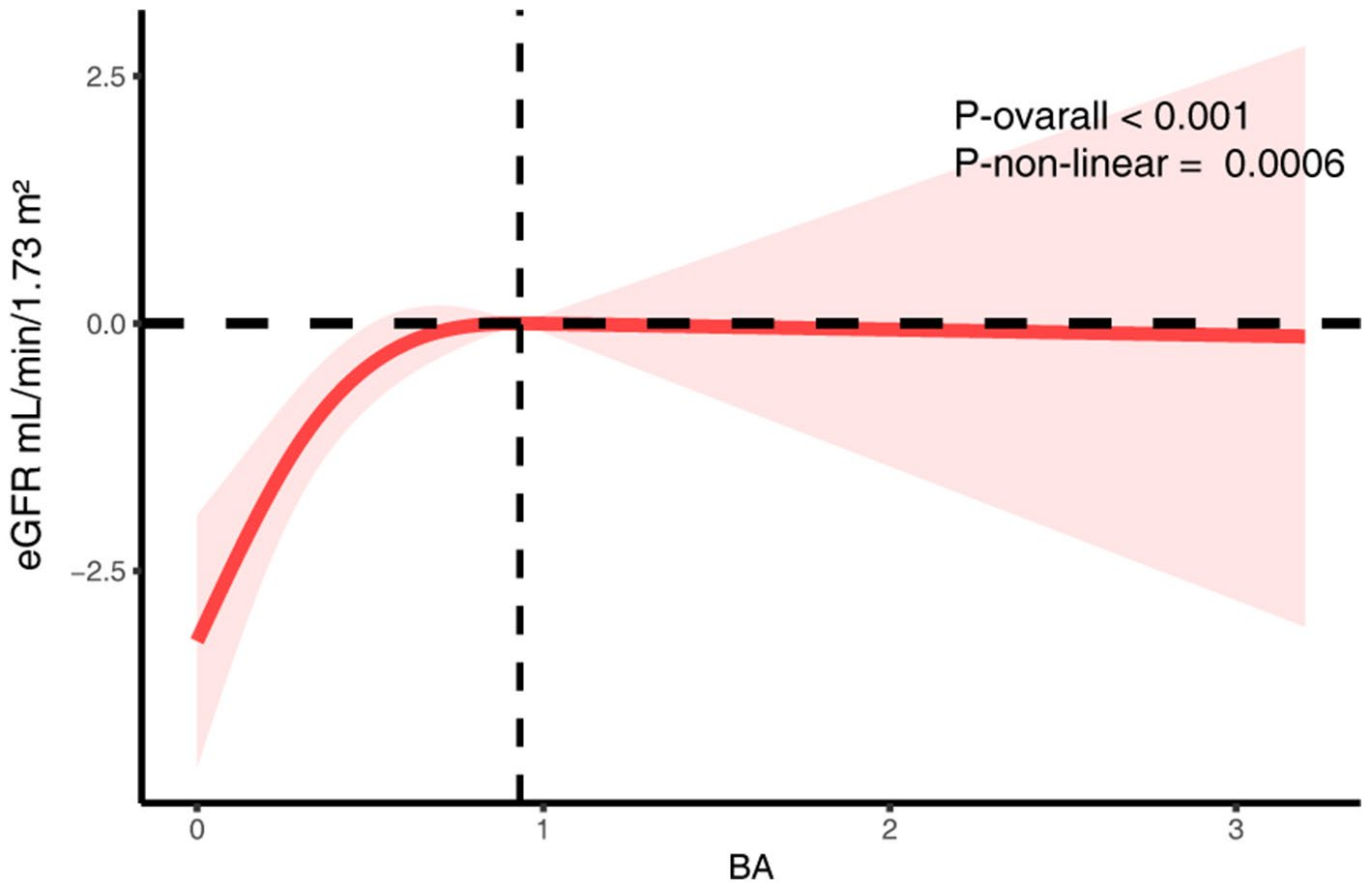
The RCS analysis of the association between dietary butyrate and eGFR. The model was adjusted for age, sex, race/ethnicity, marital status, education, BMI, alcohol user, smoke, hypertension, hyperlipidemia.

Multivariable linear regression revealed a consistent and statistically significant positive association between continuous dietary butyrate intake and eGFR across all models. In the fully adjusted model (Model 2), each 1-g increase in butyrate intake corresponded to a 1.61 mL/min/1.73 m^2^ increase in eGFR (*β* = 1.61; 95% CI: 0.29–2.92; *p* = 0.02) (Table 3). When categorized by quartiles, individuals in the highest quartile (Q4) had significantly higher eGFR compared to those in the lowest (Q1) (*β* = 2.45; 95% CI: 0.43–4.47; *p* = 0.02), with a clear linear trend (P for trend = 0.002). RCS analysis further supported a significant overall association (*p* < 0.001) and revealed a non-linear pattern (*P* for non-linearity = 0.0006), showing that the eGFR increase was most pronounced at lower butyrate intake levels and plateaued thereafter (Figure 2).

**Table 3:** Association between dietary butyrate intake and eGFR in the NHANES cohort.

	Crude model		Model 1		Model 2	
	*β* (95%CI)	*p* value	*β* (95%CI)	*p* value	*β* (95%CI)	*p* value
Continuous	4.09 (2.65, 5.53)	<0.0001	1.62 (0.31, 2.93)	0.02	1.61 (0.29, 2.92)	0.02
Q1	ref		ref		ref	
Q2	−0.52 (−2.55, 1.50)	0.61	0.1 (−1.88, 2.08)	0.92	0.19 (−1.79, 2.18)	0.85
Q3	3.48 (1.30, 5.67)	0.002	3.38 (1.51, 5.26)	<0.001	3.26 (1.42, 5.11)	<0.001
Q4	4.39 (2.09, 6.69)	<0.001	2.45 (0.42, 4.47)	0.02	2.45 (0.43, 4.47)	0.02
*P* for trend		<0.0001		0.002		0.002

Low exposure (Q1) was used as the reference group.

Model 1: race/ethnicity, age, sex, education, marital status, BMI, alcohol user, smoke.

Model 2: race/ethnicity, age, sex, education, marital status, BMI, alcohol user, smoke, hypertension, hyperlipidemia.

#### 3.2.2 Association with proteinuria

**Figure 3:**
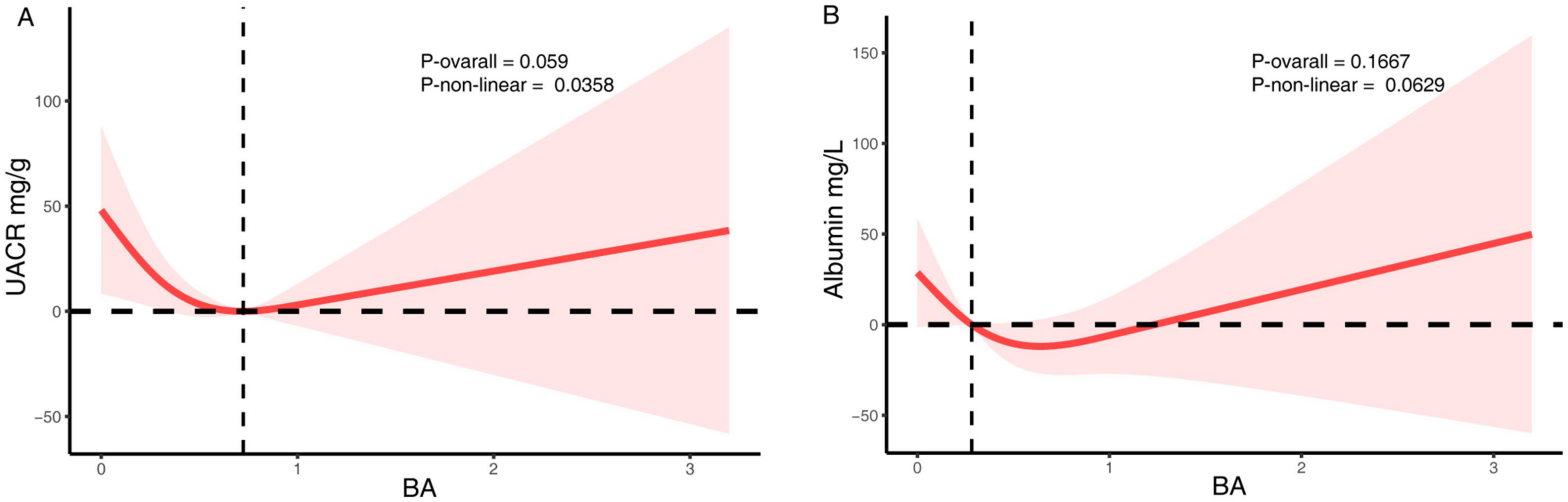
The RCS analysis of the association between dietary butyrate and proteinuria. **(A)** UACR. **(B)** Urinary albumin. The model was adjusted for age, sex, race/ethnicity, marital status, education, BMI, alcohol user, smoke, hypertension, hyperlipidemia.

In contrast, no significant linear association was found between dietary butyrate intake and UACR (Model 2: *β* = −15.12; 95% CI: −42.10 to 11.86; *p* = 0.27) (Table 4). In the quartile analyses, participants in Q3 had significantly lower UACR compared to Q1 (*p* = 0.04), but this effect was not sustained in Q4, and no consistent linear trend was observed (*P* for trend = 0.11). RCS modeling indicated a potential non-linear relationship (*P* for non-linearity = 0.04), although the overall association was not statistically significant (*p* = 0.059) (Figure 3). A sensitivity analysis examining urinary albumin concentration as an alternative proteinuria measure found no significant associations in any model (Supplementary Table S1 and Supplementary Figure S2).

**Table 4:** Association between dietary butyrate intake and UACR in the NHANES cohort.

	Crude model		Model 1		Model2	
	*β* (95%CI)	*p* value	*β* (95%CI)	*p* value	*β* (95%CI)	*p* value
Continuous	−20.79 (−52.06, 10.47)	0.19	−15.49 (−42.84, 11.86)	0.26	−15.12 (−42.10, 11.86)	0.27
Q1	ref		ref		ref	
Q2	−26.36 (−71.83, 19.10)	0.25	−28.23(−75.66, 19.20)	0.24	−30.58 (−77.49, 16.33)	0.20
Q3	−56 (−100.61, −11.39)	0.01	−49.37 (−94.32, −4.43)	0.03	−47.47 (−92.14, −2.80)	0.04
Q4	−53.16 (−100.97, −5.36)	0.03	−36.71 (−84.03, 10.61)	0.13	−36.9 (−83.89, 10.09)	0.12
*P* for trend		0.02		0.1		0.11

Low exposure (Q1) was used as the reference group.

Model 1: race/ethnicity, age, sex, education, marital status, BMI, alcohol user, smoke.

Model 2: race/ethnicity, age, sex, education, marital status, BMI, alcohol user, smoke, hypertension, hyperlipidemia.

#### 3.2.3 Subgroup analyses

**Figure 4:**
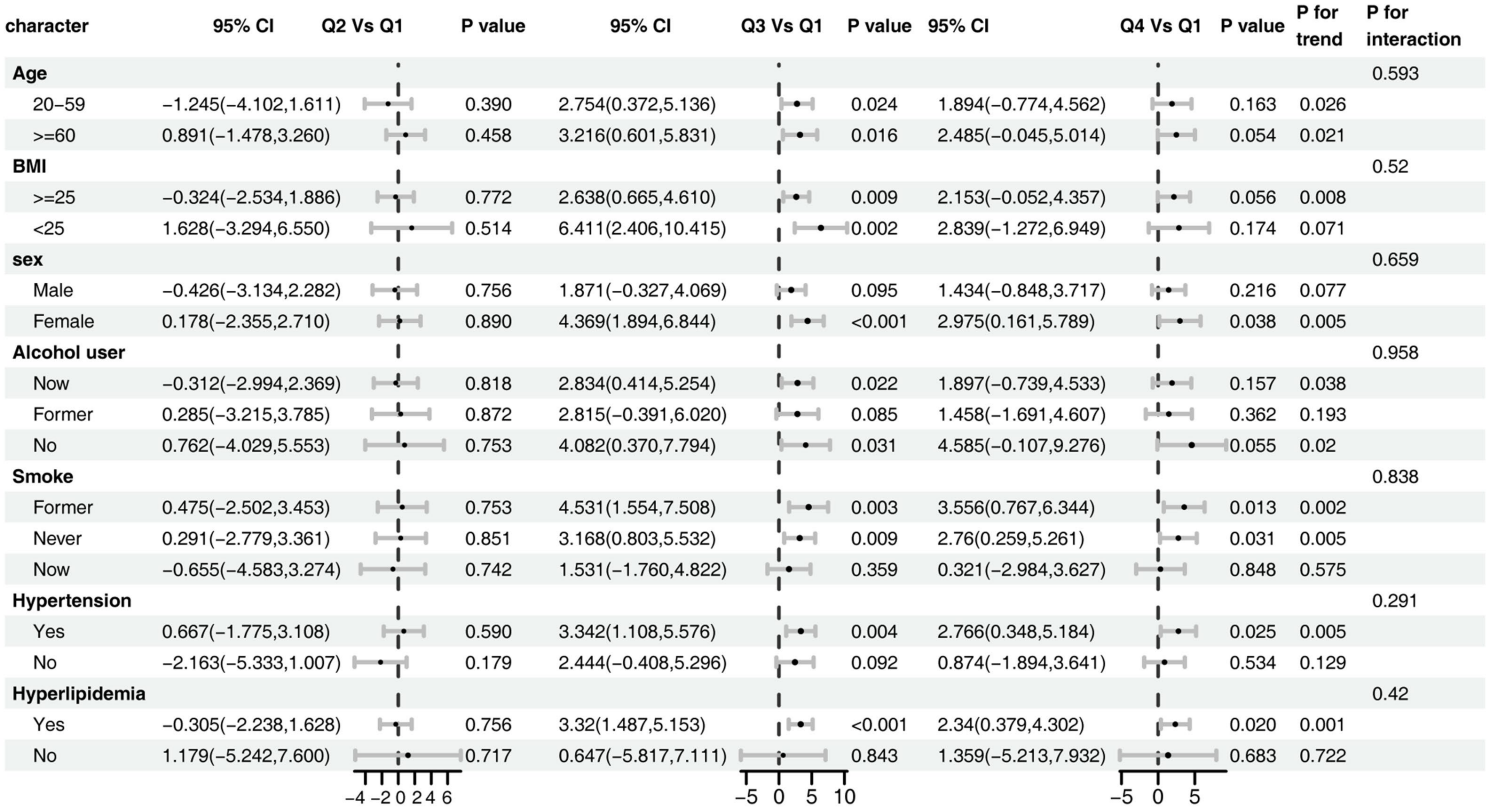
Subgroup analysis of the association between dietary butyrate and eGFR. The model was adjusted for age, sex, race/ethnicity, marital status, education, BMI, alcohol user, smoke, hypertension, hyperlipidemia.

Subgroup analyses demonstrated that the positive association between dietary butyrate and eGFR was consistent across all predefined subgroups (Figure 4). No significant interactions were observed for age, sex, BMI, smoking, alcohol consumption, hypertension, or hyperlipidemia (all *P* for interaction > 0.20), although the magnitude of the association appeared more pronounced among individuals with hypertension or hyperlipidemia.

### 3.3 Serum butyrate and isobutyrate in relation to renal function: Chinese cohort

#### 3.3.1 Association of serum butyrate with renal function

**Figure 5:**
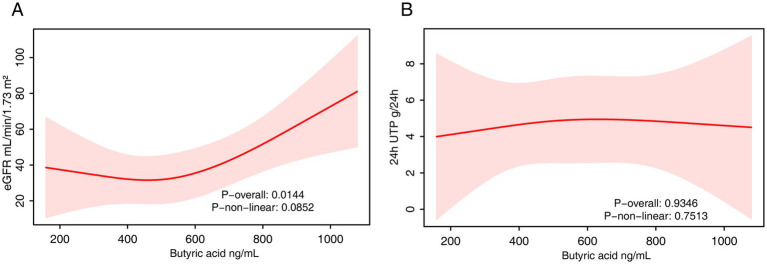
The RCS analysis of the association between serum butyrate and renal function. **(A)** eGFR. **(B)** 24-h urinary protein. The model was adjusted for age, sex and fasting plasma glucose.

In the fully adjusted model, serum butyrate levels were significantly and positively associated with eGFR (*β* = 0.05; 95% CI: 0.01–0.08; *p* = 0.02) (Table 5). However, in the quartile analysis, this association did not reach statistical significance, and no linear trend was detected (*P* for trend = 0.24). RCS analysis confirmed a significant overall association (*p* = 0.014), with no evidence of non-linearity (*p* = 0.085), suggesting a primarily linear positive relationship (Figure 5A).

**Table 5:** Association of serum butyrate concentrations with eGFR and 24-h urinary protein in the Chinese cohort.

		Crude model		Model 1		Model2	
		*β* (95%CI)	*p* value	*β* (95%CI)	*p* value	*β* (95%CI)	*p* value
eGFR	Continuous	0.05 (0.01, 0.09)	0.01	0.04 (0.00, 0.08)	0.05	0.05 (0.01, 0.08)	0.02
	Q1	ref		ref		ref	
	Q2	8.73 (−13.24, 30.70)	0.43	9.6 (−12.02, 31.22)	0.38	9 (−11.49, 29.48)	0.38
	Q3	1.05 (−20.34, 22.43)	0.92	−2.54 (−23.79, 18.71)	0.81	−3.08 (−23.29, 17.13)	0.76
	Q4	21.57 (−0.39, 43.54)	0.05	17.34 (−4.61, 39.28)	0.12	17.28 (−3.64, 38.19)	0.10
	*P* for trend		0.11		0.26		0.24
24hUTP	Continuous	0 (−0.01, 0.00)	0.44	0 (−0.01, 0.01)	0.78	0 (−0.01, 0.01)	0.85
	Q1	ref		ref		ref	
	Q2	−3.18 (−6.70, 0.33)	0.08	−3.2 (−6.36, −0.05)	0.05	−3.24 (−6.38, −0.09)	0.04
	Q3	−1.22 (−4.74, 2.29)	0.49	−0.43 (−3.61, 2.74)	0.79	−0.42 (−3.59, 2.74)	0.79
	Q4	−1.76 (−5.27, 1.76)	0.32	−0.25 (−3.48, 2.99)	0.88	−0.28 (−3.51, 2.94)	0.86
	*P* for trend		0.58		0.71		0.71

Low exposure (Q1) was used as the reference group.

Model 1: age, sex.

Model 2: age, sex, fasting plasma glucose.

With respect to 24-h urinary protein, serum butyrate was not significantly associated with protein levels in the continuous model (*p* = 0.85). In quartile analysis, Q2 showed a significantly lower urinary protein compared to Q1 (*β* = −3.24; *p* = 0.04), but this trend was not observed in higher quartiles, and no linear trend was evident (*P* for trend = 0.71). RCS analysis similarly showed no significant associations (Figure 5B).

#### 3.3.2 Association of serum isobutyrate with renal function

**Figure 6:**
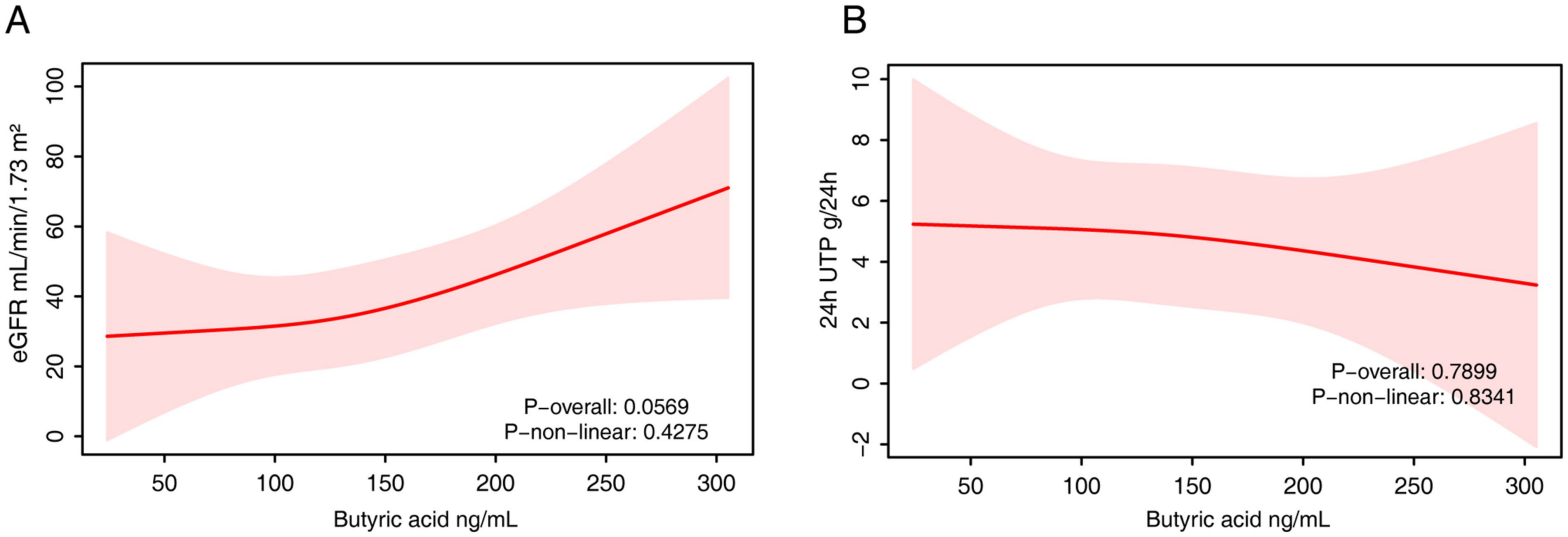
The RCS analysis of the association between serum isobutyrate and renal function. **(A)** eGFR. **(B)** 24-h urinary protein. The model was adjusted for age, sex and fasting plasma glucose.

Serum isobutyrate levels were also significantly and positively associated with eGFR in the fully adjusted model (*β* = 0.15; 95% CI: 0.02–0.28; *p* = 0.02) (Table 6). However, this association was not statistically significant in either the quartile or RCS analyses (Figure 6A). No significant associations were found between serum isobutyrate and 24-h urinary protein in any model (Table 6, Figure 6B).

**Table 6:** Association of serum isobutyrate concentrations with eGFR and 24-h urinary protein in the Chinese cohort.

		Crude model		Model 1		Model2	
		*β* (95%CI)	*p* value	*β* (95%CI)	*p* value	*β* (95%CI)	*p* value
eGFR	Continuous	0.17 (0.04, 0.31)	0.01	0.14 (0.00, 0.28)	0.05	0.15 (0.02, 0.28)	0.02
	Q1	ref		ref		ref	
	Q2	11.38 (−10.67, 33.43)	0.31	7.5 (−14.72, 29.73)	0.50	7.84 (−13.21, 28.89)	0.46
	Q3	0.75 (−20.71, 22.21)	0.94	−2.95 (−24.58,1 8.68)	0.79	−0.77 (−21.09, 19.54)	0.94
	Q4	19.79 (−2.26, 41.84)	0.08	13.51 (−9.36, 36.37)	0.24	14.56 (−6.87, 35.99)	0.18
	*P* for trend		0.18		0.44		0.32
24hUTP	Continuous	−0.02 (−0.04, 0.00)	0.11	−0.01 (−0.03, 0.01)	0.54	−0.01 (−0.03, 0.01)	0.51
	Q1	ref		ref		ref	
	Q2	−3.07 (−6.52, 0.38)	0.08	−1.97 (−5.25, 1.31)	0.23	−1.88 (−5.16, 1.41)	0.26
	Q3	−1.97 (−5.37, 1.42)	0.25	−1.05 (−4.25, 2.16)	0.52	−1.12 (−4.33, 2.09)	0.49
	Q4	−2.68 (−6.14, 0.77)	0.13	−0.85 (−4.26, 2.57)	0.62	−0.84 (−4.26, 2.57)	0.62
	*P* for trend		0.19		0.75		0.72

Low exposure (Q1) was used as the reference group.

Model 1: age, sex.

Model 2: age, sex, fasting plasma glucose.

### 3.4 Sensitivity analysis

To validate the robustness of our findings, a sensitivity analysis was conducted using a restricted NHANES subsample of participants with both diabetes and CKD (*n* = 3,049). In this subgroup, dietary butyrate intake remained significantly associated with higher eGFR in the fully adjusted model (*β* = 3.11; 95% CI: 0.33–5.89; *p* = 0.03), and a dose–response relationship was evident across quartiles (*P* for trend = 0.02), with the highest intake group demonstrating significantly higher eGFR than the lowest. Consistent with primary analyses, no significant associations were detected between dietary butyrate and UACR or urinary albumin concentration in this subgroup (Supplementary Table S2 and Supplementary Figure S3).

## 4 Discussion

By integrating data from the nationally representative NHANES cohort and an independent Chinese cohort of patients with DKD, this study is the first to systematically investigate the independent association between butyrate—a key gut microbial metabolite—and major renal function parameters in individuals with diabetes. Our principal finding is the observation that higher levels of butyrate, whether estimated from dietary intake or measured directly in serum, are significantly and independently associated with a higher eGFR. The consistency of this association across two demographically and clinically distinct populations lends robust epidemiological support to the “gut-kidney axis” hypothesis in the context of DKD and suggests that butyrate may act as a reno-protective agent.

The observed positive association between butyrate and eGFR corroborates and extends previous findings from preclinical models, thereby establishing a population-level epidemiological link between a microbial metabolite and renal function. Prior studies have explored the relationship between dietary fiber, SCFA-producing capacity, and metabolic health (25), but these often relied on indirect proxies—such as fecal SCFA concentrations or self-reported fiber intake (26)—and were generally conducted in healthy or small-sample populations. In contrast, our study includes both a large, nationally representative cohort and a clinically confirmed DKD cohort, with dual assessments of butyrate exposure through dietary estimation and serum quantification, thereby enhancing both the credibility and external validity of our findings.

Our findings, which demonstrate a population-level link between butyrate and better renal function, are strongly supported by extensive preclinical research into the gut-kidney axis in DKD. The reno-protective effects of butyrate appear to be multifaceted, addressing several core pathological mechanisms of diabetic nephropathy. The chronic hyperglycemic state in diabetes is known to induce gut dysbiosis, impairing the intestinal barrier (27) and reducing the endogenous production of SCFAs like butyrate. By serving as a primary energy source for colonocytes, butyrate is crucial for maintaining intestinal barrier integrity (28), thereby reducing the systemic translocation of pro-inflammatory bacterial products like lipopolysaccharide (LPS), which are known to exacerbate renal inflammation (29). This cascade, from intestinal dysbiosis to systemic inflammation and subsequent renal injury, exemplifies the pathological communication within the gut-kidney axis in DKD.

Mechanistically, beyond its general anti-inflammatory effects via GPR43/109A signaling (30) and immune modulation, butyrate directly counteracts key drivers of DKD progression. For instance, it has been shown to ameliorate renal oxidative stress, a central feature of hyperglycemic injury, by activating the Nrf2 signaling pathway and upregulating antioxidant enzymes (31). Furthermore, as a potent HDAC inhibitor (32), butyrate can epigenetically regulate gene expression to suppress renal fibrosis. This includes downregulating pro-fibrotic pathways such as TGF-β1/Smad signaling (33, 34), thereby inhibiting the epithelial-to-mesenchymal transition (EMT) (35) and extracellular matrix deposition that lead to glomerulosclerosis and tubular damage. This mechanistic evidence from preclinical models provides a strong biological rationale for the positive association between butyrate levels and preserved eGFR observed in our dual-cohort study.

Interestingly, we did not observe a consistent association between butyrate levels and albuminuria. Rather than undermining our main findings (18), this “negative” result may indicate that butyrate primarily exerts its effects on the tubulointerstitial compartment rather than the glomerular filtration barrier. Albuminuria is primarily a consequence of podocyte and slit diaphragm dysfunction, involving key proteins such as nephrin and podocin (36, 37), whereas butyrate and other SCFAs act predominantly on proximal tubular cells (38) and immune regulators (39). Additionally, the UACR measure used in NHANES is based on single-spot urine samples, which are subject to variability due to hydration status and circadian rhythms—potentially obscuring subtle or non-linear associations. Notably, the non-linear trend we observed could imply a “window effect,” wherein butyrate modulates glomerular permeability only within a specific concentration range, with diminishing returns beyond a threshold. Future studies incorporating single-cell transcriptomics could further explore this possibility by characterizing podocyte-specific responses to SCFA exposure.

Our findings from the Chinese DKD cohort—that serum butyrate remains positively associated with eGFR even in patients with advanced renal impairment—suggest that its protective effects may persist into intermediate or late disease stages. While direct comparison between studies is difficult due to varied analytical methods, the serum butyrate levels in our cohort are within the range reported in other studies of patients with kidney disease (40, 41). Interestingly, our analysis in this cohort also revealed a significant positive association between serum isobutyrate and eGFR. Unlike butyrate, which is derived from fiber, isobutyrate originates from the microbial fermentation of branched-chain amino acids. This finding is intriguing, as it may suggest that higher isobutyrate levels in patients with better renal function could reflect a healthier protein metabolism or a specific, beneficial gut microbial profile, rather than a direct causal effect (42, 43). Although emerging evidence points to its potential anti-inflammatory properties (44), further research is needed to determine whether isobutyrate is a direct reno-protective agent or a valuable biomarker of metabolic health in DKD. This has important implications for identifying a potential therapeutic window: it indicates that interventions aimed at increasing butyrate levels could still be effective even in individuals with substantial renal dysfunction. Clinically, although our findings are not sufficient to revise current treatment guidelines, they lend mechanistic support to existing dietary recommendations. The observed relationship between higher butyrate levels and improved renal function underscores the importance of promoting dietary patterns rich in fermentable fibers, not only for glycemic control but also for potential renal benefits via increased endogenous butyrate production (45). Butyrate may also emerge as a valuable biomarker for assessing gut-mediated renal protection and a promising target for future precision microbiome-based therapies.

The key strengths of our study include its dual-cohort design, which integrates the statistical power and generalizability of NHANES with the granular phenotyping of a clinical validation cohort. We employed rigorous statistical methodologies, including sample weighting and comprehensive adjustment for potential confounders. However, several limitations must be acknowledged. First, the cross-sectional design precludes causal inference, and reverse causality cannot be ruled out—that is, declining renal function may itself alter gut microbial composition and reduce butyrate production (46, 47). Second, dietary butyrate intake was estimated using 24-h dietary recall, which is susceptible to recall bias and may not capture long-term dietary habits. Third, the relatively small sample size and single-center nature of the Chinese cohort may limit its statistical power and external validity. Finally, despite careful adjustment for a wide range of covariates, the possibility of residual confounding cannot be excluded.

This study has several key strengths, including its dual-cohort design, which integrates the statistical power and generalizability of NHANES with the granular phenotyping of a clinical validation cohort. However, some limitations must be acknowledged. First, the cross-sectional design precludes any causal inference, and the possibility of reverse causality cannot be ruled out; that is, declining renal function may itself alter gut microbial composition and reduce butyrate production (46, 47). Second, dietary butyrate intake in the NHANES cohort was estimated using 24-h dietary recalls, which are susceptible to recall bias and may not reflect long-term dietary habits. Third, our analysis was limited by the unavailability of certain clinical data, such as the duration of diabetes or CKD and adherence to medications, which may act as residual confounders. Fourth, this study did not include a direct analysis of the gut microbiota, which prevents us from linking the observed butyrate levels to specific microbial compositions and functions. Fifth, the single-center nature and relatively small sample size of our Chinese cohort may limit its statistical power and the generalizability of the serum-based findings to other populations. Finally, despite careful adjustment for a wide range of covariates, the possibility of residual confounding from unmeasured factors cannot be entirely excluded. Future prospective studies incorporating gut microbiome data are warranted to confirm our findings and elucidate the underlying mechanisms.

## 5 Conclusion

In summary, this dual-cohort study provides compelling evidence that higher levels of butyrate are independently associated with better-preserved glomerular filtration function in individuals with diabetes. The association with albuminuria was less consistent and potentially non-linear, suggesting distinct pathophysiological pathways may underlie glomerular filtration versus permeability. While prospective studies are needed to establish causality, these findings underscore the potential relevance of the gut-kidney axis in DKD. They also highlight microbiota-targeted interventions that enhance butyrate levels as a promising avenue for renoprotection in this high-risk population.

## Data Availability

The raw data supporting the conclusions of this article will be made available by the authors, without undue reservation.
