# Supplementary material for: Dietary and circulating butyrate are independently associated with kidney function in diabetes: a dual-cohort analysis
Source: Front Nutr. 2025 Sep 29;12:1671238. doi: 10.3389/fnut.2025.1671238 (PMC12515660; doi:10.3389/fnut.2025.1671238)
Supplement: Supplementary file 1 [file Data_Sheet_1.PDF]

Table S1. Association Between Dietary Butyrate Intake and Urinary Albumin Concentration in the NHANES Cohort

	Crude model		Model 1		Model2	
	β (95%CI)	*P* value	β (95%CI)	*P* value	β (95%CI)	*P* value
Continuous	-13.94(-49.53,21.65)	0.44	-5.58(-41.82,30.65)	0.76	-5.25(-41.13,30.63)	0.77
Q1	ref		ref		ref	
Q2	-9.82(-58.67,39.02)	0.69	-6.01(-56.88,44.86)	0.82	-8.2(-58.55,42.15)	0.75
Q3	-45.77(-89.26,-2.28)	0.04	-36.67(-79.06,5.73)	0.09	-34.64(-76.79,7.50)	0.11
Q4	-36.52(-83.41,10.37)	0.13	-18.15(-64.89,28.58)	0.44	-18.29(-64.82,28.25)	0.44
P for trend		0.07		0.3		0.32

Low exposure (Q1) was used as the reference group.

model 1: race/ethnicity, age, sex, education, marital status, BMI, alcohol user, smoke

model 2: race/ethnicity, age, sex, education, marital status, BMI, alcohol user, smoke, hypertension, hyperlipidemia

Table S2. Association Between Dietary Butyrate Intake and Renal Function Among Participants with Diabetes and CKD in NHANES

		Crude model		Model 1		Model 2	
		Beta(95%CI)	*P* value	Beta(95%CI)	*P* value	Beta(95%CI)	*P* value
eGFR	Continuous	8.43(5.20,11.67)	<0.0001	3.23(0.38,6.07)	0.03	3.11(0.33,5.89)	0.03
	Q1	ref		ref		ref	
	Q2	0.16(-3.79,4.11)	0.94	1.88(-1.80,5.55)	0.31	1.81(-1.82,5.44)	0.33
	Q3	3.17(-0.66,6.99)	0.10	3.85(0.62,7.09)	0.02	3.83(0.56,7.10)	0.02
	Q4	8.38(4.10,12.66)	<0.001	4.73(0.64,8.83)	0.02	4.61(0.57,8.66)	0.03
	P for trend		<0.0001		0.01		0.02
UACR	Continuous	-21.38(-119.11,76.35)	0.67	-49.86(-141.16,41.44)	0.28	-44.53(-133.74,44.68)	0.33
Urinary albumin	Continuous	0.77(-111.99,113.52)	0.99	-19.58(-136.73,97.56)	0.74	-14.05(-129.16,101.06)	0.81

Low exposure (Q1) was used as the reference group.

model 1: race/ethnicity, age, sex, education, marital status, BMI, alcohol user, smoke

model 2: race/ethnicity, age, sex, education, marital status, BMI, alcohol user, smoke, hypertension, hyperlipidemia

Supplementary Figure S1. Representative UPLC-MS/MS Chromatograms.

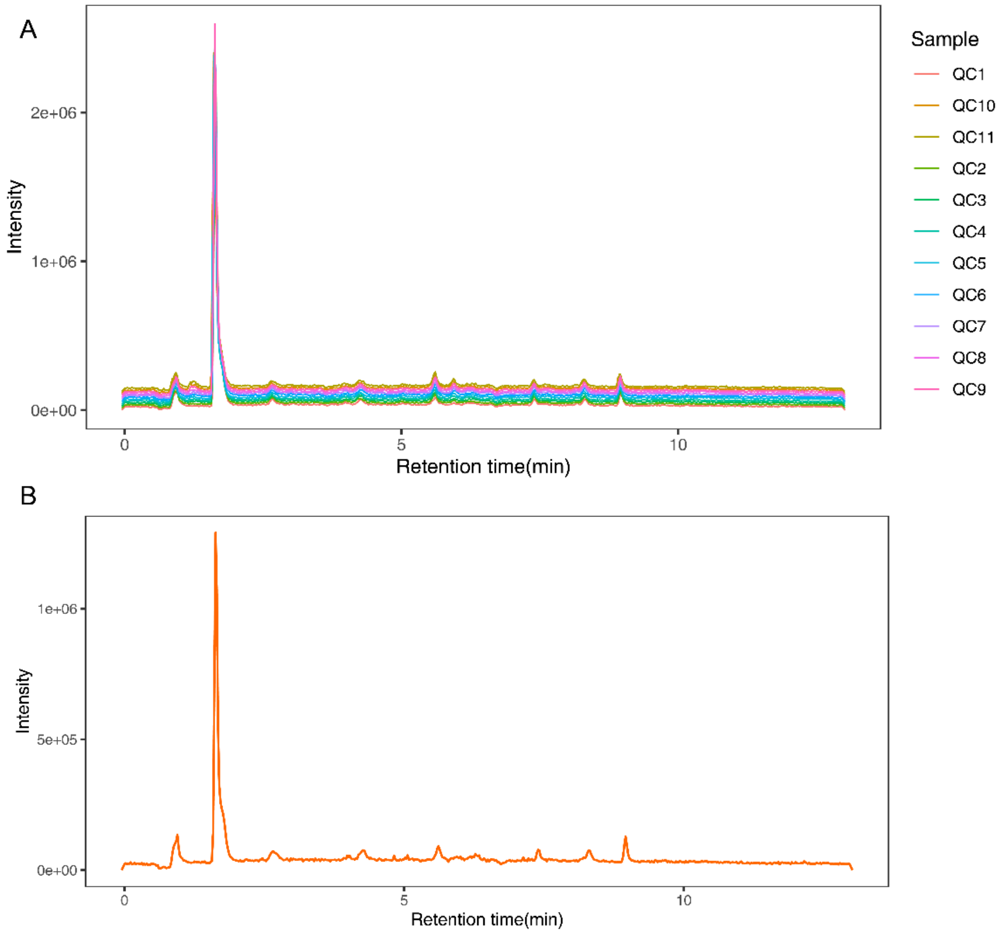

(A) An overlay of chromatograms from all Quality Control (QC) samples interspersed throughout the analytical run. The high degree of signal overlap among the QC samples demonstrates the stability, robustness, and reproducibility of the UPLC-MS/MS analytical platform. (B) A representative Total Ion Chromatogram (TIC) of a single serum sample from the Chinese DKD cohort, showing the overall metabolic profile.

Figure S2. Restricted Cubic Spline Analysis of the Association Between Dietary Butyrate Intake and Urinary Albumin Concentration in the NHANES Cohort

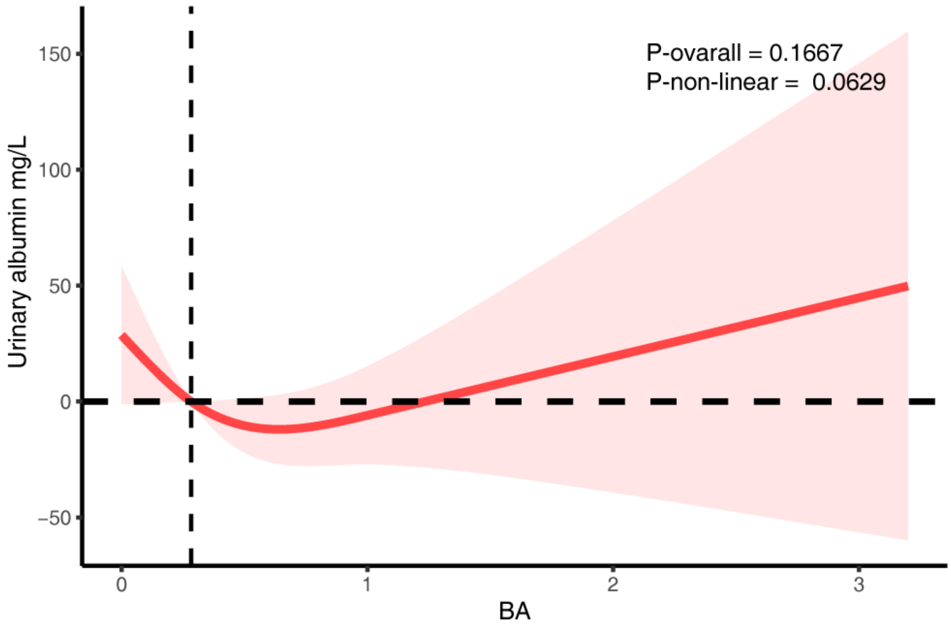

The model was adjusted for age, sex, race/ethnicity, marital status, education, BMI, alcohol user, smoke, hypertension, hyperlipidemia.

Figure S3. Association Between Dietary Butyrate Intake and Renal Function in Adults with Diabetic Kidney Disease: Dose–Response and Subgroup Analyses

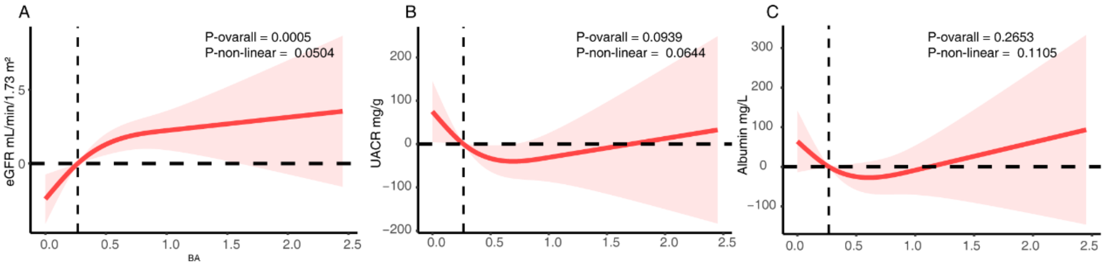

D

character	95% CI	Q2 Vs Q1	P value	95% CI	Q3 Vs Q1	P value	95% CI	Q4 Vs Q1	P value	P for trend	P for interaction
**Age**											0.035
20–59	−0.338(−7.089,6.413)		0.921	3.098(−2.827,9.022)		0.302	8.658(1.945,15.372)		0.012	0.004	
60–85	1.375(−2.683,5.434)		0.504	3.136(−0.804,7.077)		0.118	1.413(−2.952,5.779)		0.523	0.428	
**BMI**											0.355
>=25	1.177(−2.851,5.205)		0.564	2.531(−0.909,5.970)		0.148	4.157(−0.128,8.442)		0.057	0.041	
<25	0.92(−6.637,8.476)		0.809	9.72(2.184,17.257)		0.012	5.602(−1.774,12.979)		0.134	0.049	
**Sex**											0.847
Male	−0.192(−4.390,4.007)		0.928	2.613(−0.975,6.201)		0.152	2.639(−1.253,6.531)		0.182	0.109	
Female	1.711(−3.753,7.175)		0.537	3.853(−0.884,8.589)		0.110	6.021(−0.357,12.399)		0.064	0.042	
**Alcohol user**											0.876
Yes	0.727(−4.995,6.450)		0.802	2.363(−2.411,7.137)		0.329	4.541(−1.077,10.158)		0.112	0.068	
Former	2.139(−3.756,8.034)		0.474	4.16(−2.065,10.385)		0.188	2.218(−3.343,7.779)		0.431	0.384	
No	1.694(−6.778,10.167)		0.692	4.94(−1.024,10.904)		0.103	8.085(−0.255,16.426)		0.057	0.034	
**Smoke**											0.979
Former	3.023(−2.282,8.328)		0.262	4.004(−1.328,9.336)		0.140	4.31(−1.737,10.357)		0.161	0.173	
Never	0.458(−5.319,6.236)		0.876	3.439(−0.953,7.831)		0.124	4.997(0.173,9.822)		0.042	0.016	
Now	2.695(−5.520,10.910)		0.515	5.173(−2.209,12.556)		0.167	5.121(−1.744,11.985)		0.141	0.092	
**Hypertension**											0.206
Yes	2.073(−2.091,6.237)		0.327	3.112(−0.533,6.757)		0.094	4.437(0.111,8.763)		0.044	0.038	
No	−4.585(−11.474,2.305)		0.190	3.832(−1.500,9.164)		0.157	2.432(−3.200,8.065)		0.393	0.098	
**Hyperlipidemia**											0.296
Yes	0.5(−3.191,4.191)		0.789	2.976(−0.355,6.307)		0.080	3.247(−0.844,7.337)		0.119	0.067	
No	7.577(−3.804,18.958)		0.188	8.994(−2.057,20.046)		0.109	13.725(0.704,26.747)		0.039	0.034	
		−10 0 10			0 5 10 15 20			0 10 20			

The RCS and subgroup analyses of the association between dietary butyrate and renal function in participants with both diabetes and CKD. (A) eGFR; (B) UACR; (C) Urinary albumin. RCS models were adjusted for age, sex, race/ethnicity, marital status, education, BMI, alcohol use, smoking status, hypertension, and hyperlipidemia. (D) Subgroup analysis of the association between dietary butyrate intake and eGFR by age, sex, BMI, smoking, alcohol use, hypertension, and hyperlipidemia. The model was adjusted for age, sex, race/ethnicity, marital status, education, BMI, alcohol user, smoke, hypertension, hyperlipidemia.
